# The protective effect of higher serum TAG (51:4) levels against Parkinson’s disease

**DOI:** 10.1017/S0007114524002137

**Published:** 2025-03-28

**Authors:** Yajun Jing, Jinye Su, Honglin Zhu, Yiming Chen, Surmai Shukla, Lianghong Yu, Dengliang Wang, Dezhi Kang

**Affiliations:** 1 Department of Neurosurgery, Neurosurgery Research Institute, The First Affiliated Hospital, Fujian Medical University, Fuzhou 350005, Fujian, People’s Republic of China; 2 Faculty of Life Sciences and Medicine, King’s College London, London, UK; 3 Department of Medical Laboratory, College of Medical Technology and Engineering, Fujian Medical University, Fuzhou, Taijiang District, Fuzhou, Fujian 350004, People’s Republic of China; 4 Department of Immunology, Northwestern University, Evanston, Illinois, USA; 5 Department of Neurosurgery, National Regional Medical Center, Binhai Campus of the First Affiliated Hospital, Fujian Medical University, Fuzhou 350212, Fujian, China; 6 Fujian Provincial Institutes of Brain Disorders and Brain Sciences, First Affiliated Hospital, Fujian Medical University, Fuzhou 350005, Fujian, People’s Republic of China; 7 Institute of Neurology, First Affiliated Hospital, Fujian Medical University, Fuzhou 350005, Fujian, People’s Republic of China

**Keywords:** Lipid metabolism, Parkinson’s disease, Circulating inflammatory proteins, Circulating immune cells, Mendelian randomisation analysis

## Abstract

Emerging evidence has shown a strong correlation between serum TAG levels, the inflammatory response and Parkinson’s disease (PD) onset. However, the causal relationship between TAG levels and PD has not been well established. We aimed to investigate the relationship between serum TAG levels and risk of PD and explore the potential mediating role of circulating immune cells and inflammatory proteins. We utilised genotype data from the GeneRISK cohort, and summary data from genome-wide association studies investigating PD, circulating immune cells, inflammatory proteins and plasma lipidomes. Using Mendelian randomisation (MR) and multivariate MR (MVMR) analysis, we further adjusted for phosphatidylcholine (17:0_18:1) and TAG (58:7). Our results suggested a robust causal link between higher serum TAG (51:4) levels and a decreased risk of PD, with 1 sd genetically instrumented higher serum TAG (51:4) level leading to a 21 per cent (95 % CI 0·66, 0·96) reduction in the risk of PD (*P*= 0·015). Additionally, the results of the mediation analysis suggested a possible role for mediation through circulating immune cells (including IgD-CD38-B cells and resting CD4 regulatory T cells), but not circulating inflammatory proteins, in the causal relationship between the plasma lipidomes and PD. Our study confirms a causal relationship between higher serum TAG (51:4) levels and a lower risk of PD and clarifies a possible role for mediation through circulating immune cells, but not inflammatory proteins. These findings indicate that serum TAG (51:4) regulates immunity to effectively lower the risk of PD.

Parkinson’s disease (PD) is one of the most prevalent neurodegenerative disorders and is primarily characterised by tremors, increased muscle rigidity, bradykinesia, and impaired gait and balance, collectively known as Parkinsonism^(1,2)^. Currently, PD constitutes one of the leading causes of disability globally, imposing substantial economic and social burdens^(1,3)^. According to epidemiological reports, PD affects 1 % of individuals over the age of 60 years, and its incidence varies from one to two cases per 1000 people in unselected groups^(3,4)^. Notably, PD is uncommon before the age of 50 years, and its prevalence increases to 4 % in older age groups^(5)^. At autopsy, *α*-synuclein (*α*-syn) positive protein clumps (Lewy bodies) and the degeneration of dopaminergic neurons in the substantia nigra pars compacta are the two hallmarks of PD^(6)^. Several hypotheses have been proposed to explain the neurodegeneration observed in PD, including oxidative stress, mitochondrial dysfunction, lysosomal dysfunction and inflammation,^(7–10)^. Although the precise aetiology of PD remains unclear, emerging genetic data have confirmed that PD is caused by a complex interplay between age, genetic predisposition and environmental exposure^(11,12)^.

Among all bodily tissues, the brain has one of the highest lipid concentrations. It contains a wide range of lipids, including fatty acids, TAG, phospholipids, sterols and glycolipids^(13)^. Large-scale high-throughput sequencing has identified several PD risk genes associated with lipid metabolism^(14)^. Specifically, the most prevalent genetic variables that raise the risk of PD are missense mutations in GBA1, which encodes the lysosomal hydrolase glucocerebrosidase^(14,15)^. Additionally, PD risk is associated with mutations in SMPD1, which encodes an acid, sphingomyelinase^(16)^. Previous evidence has shown that polymorphisms in GALC and ASAH1, which encode lysosomal enzymes that catabolise sphingolipids, also increase the risk of PD^(17,18)^. Other lipid-related genes associated with an increased risk of PD include DGKQ (encoding diacylglycerol kinase theta)^(19,20)^, which is crucial for the formation of synaptic vesicles by mediating the regeneration of phosphatidylinositol from diacylglycerol^(21,22)^, and SREBF1, which encodes sterol regulatory element binding transcription factor 1^(23)^, essential for biosynthesis and cell membrane integrity^(24)^. Interestingly, new high-resolution histochemical methods have shown that Lewy bodies comprise membrane lipids, mitochondria, lysosomes and *α*-syn fibrils, among other cellular components^(25)^, indicating that the functions of lipids in biomembranes are vital in both physiological and pathological contexts of *α*-syn^(26)^. In addition, the identification of ASAH1, GBA1 and GALC as genetic risk factors for PD with substantial Lewy body deposition reinforces the hypothesis that lipids play a role in the aggregation and propagation of *α*-syn.

TAG, the predominant dietary lipids found in fats and vegetable oils (such as olive oil), are neutral lipids consisting of a glycerol backbone attached to three fatty acyl chains^(27)^. As the primary energy storage components in a variety of species, including algae, mammals and oleaginous bacteria, TAG are essential for cellular energy balance, lipid homoeostasis, development and maintenance^(28)^. In humans, most (and possibly all) cell types synthesise TAG. Abnormalities in TAG levels have been linked to several diseases, including obesity and CVD. Evidence indicates that obesity and metabolic syndrome are linked to high levels of TAG (hypertriacylglycerolaemia) and that the release of TAG from adipose tissue may contribute to cachexia, a multi-organ wasting disease^(29–31)^. Recent longitudinal studies have demonstrated a relationship between midlife blood TAG levels and the risk of cognitive decline in older adults^(32,33)^. Tan *et al.* demonstrated^(34)^ that moderate cognitive impairment in patients with PD is linked to elevated blood TAG levels. However, Zhang *et al.* found an association between reduced serum TAG levels and worse motor performance in patients with PD^(35)^. Therefore, the association between blood TAG levels and PD is complicated by conflicting findings from various studies.

Well-powered genome-wide association studies (GWAS) have identified hundreds of SNP associated with PD, serum lipids, circulating immune cells and circulating inflammatory protein levels, presenting an opportunity to test the genetic relationships between a series of risk factors and PD using Mendelian randomisation (MR) analysis. The causal association between serum TAG levels and the risk of PD remains unknown. Therefore, in this study, we examined the potential causal relationship between serum TAG levels and PD risk using MR analysis. Additionally, a range of lipids, such as sterols, fatty acids and their metabolites, complex lipids (such as glycerophospholipids and sphingolipids), and lipoproteins, have been found to have immunomodulatory and pro-/anti-inflammatory properties^(36,37)^. Chronic neuroinflammation plays a major role in dopaminergic neuron degeneration. Thus, our study examined the causal relationship between plasma lipidomes and PD, related primarily to serum TAG (51:4) levels and attempted to explore the possible role of mediation through circulating immune cells or inflammatory proteins.

## Methods

### Traits analysed and genome-wide association studies

The GWAS summary meta-analysis for PD, circulating immune cells, inflammatory proteins and the plasma lipidome have been made publicly available. The largest meta-analysis of individuals of European ancestry currently available provides genetic association data for the plasma lipidome^(38)^. Briefly, we employed genotype data at the summary level from the prospective GeneRISK cohort, which examined genome-wide correlations for 179 lipid species among 7174 participants. Genetic information for circulating immune cells was derived from GWAS summary statistics from Orru *et al.* (2022)^(39)^, which included a total of 731 immunophenotypes across four categories: absolute cell counts (*n* 118), median fluorescence intensities reflecting surface antigen levels (*n* 389), morphological parameters (*n* 32) and relative cell counts (*n* 192). GWAS data on circulating inflammatory proteins were obtained from a GWAS project and assessed in eleven cohorts of 14 824 people of European ancestry using the Olink Target Inflammation Panel^(40)^. The genetic details of PD were derived from a cross-population map of genetic correlations^(41)^. In short, we employed the findings from an inverse variance meta-analysis limited to individuals of European and East Asian ancestry, taking into account age, sex and the main components representing ancestry (2978 cases; 635 168 controls).

### Mendelian randomisation analyses

**Figure 1.**
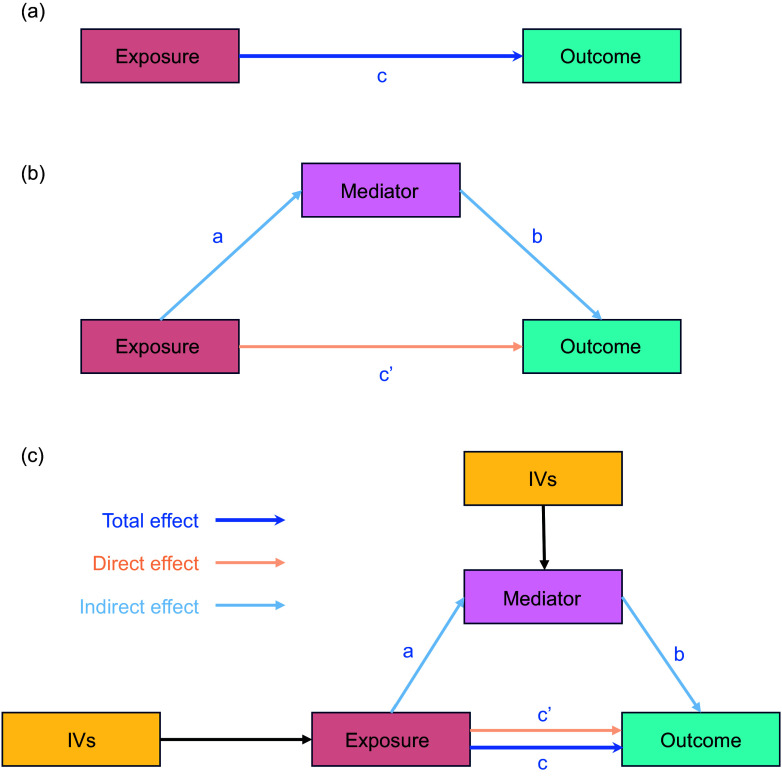
MR analysis diagram. The top half represents the unmediated causal relationship between exposure (X) and outcome (Y) (a). Path *c* represents the total effect. In the bottom half of the diagram, a third variable (M), the mediator, has been included to explain the relationship between X and Y, in the sense that part or all the effects of X on Y are channelled through M. The coefficient *a* indicates the effect of X on M, *b* indicates the effect of M on Y and *c’* is the unique effect of X on Y after M has been controlled for. The latter is known to have a direct effect. The indirect effect was defined as product *ab* (b) and (c). MR, Mendelian randomisation; IVW, inverse variance-weighted.

GWAS summary statistics were used to conduct bidirectional two-sample MR, with α *L*ipids→PD representing the causal impact of lipids (exposure) on PD (outcome) and α PD→*L*ipids representing the causal impact of PD (exposure) on lipids (outcome) (Fig. 1). Harmonised SNP significantly associated (p < 5e-8) with the exposure were clumped (*p*1 = 0·0001, *p*2 = 0·01, *kb* = 10 000 and *r*2 = 0·01) with PLINK v1.9^(42)^ and retained as instrumental variables (IV). Due to the complex long-range linkage disequilibrium structure of the HLA locus, SNP mapping to that region (chr6:25 000 000–37 000 000; GRCh37/hg19) were also excluded from our IV^(43)^. For each exposure–outcome pair, further IV were removed based on differences in allele frequency (≥ 0·05) and Steiger filtering (*Z* ≤ –1·96). Bidirectional MR analyses were carried out with the TwoSampleMR R package (v0.5.7)^(44)^, primarily through the inverse variance-weighted (IVW) method. Additionally, MR methods that were complementary were used. We employed MR-Egger regression, which is generally regarded as conservative in the presence of pleiotropic variants and less likely to produce inflated test statistics leading to false-positive associations^(45–47)^. The weighted median estimator was also used for its benefit in producing valid estimates if at least 50 % of the information in the analysis comes from SNP that are valid IV^(45–47)^. Ultimately, the weighted mode-based estimation approach was employed, which yields the most robust estimates in cases where the majority of invalid instruments nevertheless yield a consistent estimate of the real causal impact.

### Mediation analysis

Two-step MR (also known as network MR) is similar to the product of coefficient methods. Two-step MR estimates were calculated to find: (i) the causal effect of exposure on the mediator and (ii) the causal effect of mediator on the outcome (Fig. 1(c)). We used two-step MR for mediation when we found the following (1) evidence to support an effect of the trait on circulating immune cells and inflammatory proteins (step 1); (2) limited evidence to support an effect of circulating immune cells and inflammatory proteins on the trait; and (3) evidence of an effect on circulating immune cells and inflammatory proteins on PD (step 2). An arbitrary false discovery rate of 5 %, calculated according to the Benjamini–Hochberg method, was used as an indicator of supportive evidence for an association^(48)^. For the indirect effect of the mediator of the two-step MR for mediation, we multiplied the estimate of the effect of the trait on circulating immune cells and inflammatory protein levels obtained from the univariate MR by the effect of the circulating immune cells and inflammatory protein levels on PD obtained from the univariate MR. Additionally, total effect was defined as the effect of the exposure on the outcome through all potential pathways, or the total effect was the effect of exposure on the outcome without any mediator (Fig. 1(a)).

### Multivariate Mendelian randomisation analyses

Multivariate MR (MVMR) regression estimates were compared with the univariate MR estimates. For the univariate MR, we either used the same IV as in the MVMR or employed a subset of IV that were retained after Steiger filtering between both the outcome and the exposure of interest, as well as between the exposure of interest and other exposures. There was weak instrument bias using conditional F-statistics^(49)^ and heterogeneity using Cochran’s Q-statistic^(50)^.

### Selection of instrumental variables and sensitivity analyses

**Figure 2.**
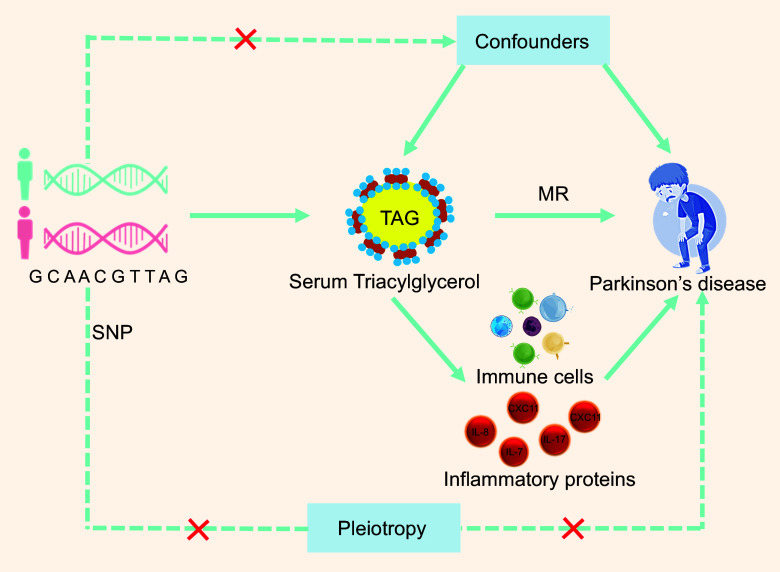
The three key assumptions of Mendelian randomisation (MR) studies. Depicted in this illustration are three assumptions of MR. The relevance assumption can be readily verified as long as the F-statistic for the SNP–exposure association exceeds 10. It is important to ensure that the correlations with established confounders are null, although the independence assumption is difficult to verify because of potential issues with pleiotropy and population substructure. Exclusion restriction assumptions are generally difficult to verify given that SNP may have pleiotropic effects or may be correlated with genes in LD that affect outcomes regardless of exposure. LD, linkage disequilibrium; IV, instrumental variables.

In our analyses, only IV that met the relevance, independence and exclusion restriction assumptions satisfied the three MR assumptions (Fig. 2). The validity of the first MR assumption was confirmed by identifying a significant association between all exposure IV used in the subsequent sensitivity analyses and exposure to MR at *P*< 5E-8. Additionally, we verified the accuracy of the second MR assumption by carefully choosing independent variables with a linkage disequilibrium score of less than 0·01 after clumping at a 1000 kb range. Subsequently, to assess the robustness of the results, sensitivity studies for associations with significant IVW MR effects were conducted using alternative MR methods such as MR-Egger, simple mode, weighted median and weighted mode. Cochran’s Q-statistics were used to evaluate heterogeneity. Additionally, we ran MR-PRESSO^(51)^ for relationships with significant IVW MR effects, given a high fraction of raised Q-statistics. To confirm that our findings were not influenced by pleiotropy, which violates the MR assumption that exposure alone influences the outcome^(52)^, we first filtered genome-wide significant exposure SNP and harmonised these SNP with the available GWAS summary statistics, confirming that SNP were present in the summary statistics of all traits. This step was completed prior to clumping to ensure that the identified IV were consistently present across all outcomes and allowed for future comparisons. To ensure that the selected SNP were more strongly connected with the exposure than with any of the other identified characteristics, Steiger filtering was used between the trait and exposure after clustering. SNP that passed all trait filtering were kept as IV and MR studies were performed on them. Considering the diversity of our phenotypes, this method served as an effective pleiotropic filter. We know that no overlap can result in the ‘winner’s curse’ bias, even while sample overlap in two-sample MR may skew results towards observational effects^(53,54)^. Weak instruments tend to exacerbate this bias. We employed MR-APSS^(55)^ (default settings and linkage disequilibrium scores from 1000 Genomes Data^(56)^), which handles both sample overlap and pleiotropy; however, thorough simulations have shown that both problems result in mild bias^(54)^.

### Statistical analysis

Two-sample MR analyses were used to estimate the causal effects of the risk factors related to serum TAG levels in patients with PD. The IVW method was selected as the primary analysis to meta-analyse individual Wald-type ratios of IV under a random-effects model, with OR described per s d increase in the risk factor level. TwoSampleMR (version 0.5.6), MR-cML (version 0.0.0.9) and MR-PRESSO (version 1.0) packages implemented in R (version 3.4) were used for analyses. Forest plots were constructed using the ggplot2 package (version 2.0.1).

## Results

### Association of serum TAG (51:4) levels with Parkinson’s disease

**Figure 3.**
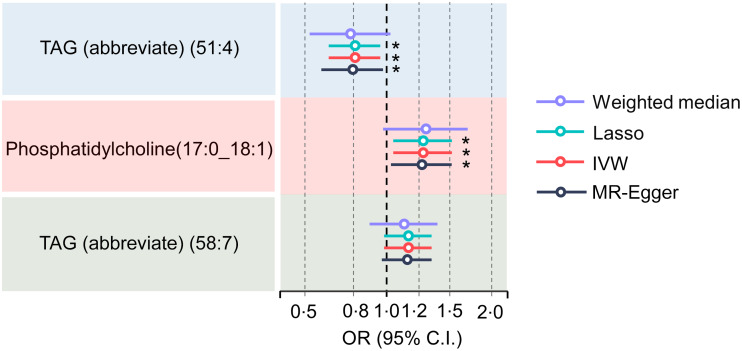
Forest plot for the results from MR analyses testing the effect of serum TAG (51:4) levels on the risk of PD. The results of the five MR analysis approaches are presented OR and 95 % CI. MR, Mendelian randomisation; PD, Parkinson’s disease; *P* values less than 0·05 are indicated by *, 0·01 and 0·001 by **, *** and *, respectively.

Initial IVW-MR revealed a significant association between elevated serum TAG levels (51:4) and a lower risk of PD. Despite the fact that other robust MR analysis approaches did not find a significant causal relationship between serum TAG (51:4) levels and the risk of PD (OR = 0·93 for the MR-Egger, *P*= 0·238; OR = 0·89 for the weighted median, *P*= 0·17; OR = 0·83 for the simple mode, *P*= 0·164; and OR = 0·85 for the weighted mode, *P*= 0·861), the direction of the results from these approaches was still consistent with the results from IVW-MR (online Supplementary Fig. S1A). Additionally, we found a significant association between increased risk of PD and increased serum phosphatidylcholine (17:0_18:1) and TAG (58:7) levels (online Supplementary Fig. S1B and C). We also found a robust relationship between higher serum TAG (51:4) levels and a lower risk of PD after further adjustment for phosphatidylcholine (17:0_18:1) and TAG (58:7) levels using MVMR. Particularly, there was a 21 % reduction in the risk of PD with every sd increase in serum TAG (51:4) levels (OR = 0·79, IVW: *P*= 0·015, OR = 0·78, MR-Egger: *P*= 0·030; OR: 0·79, Lasso: *P*= 0·016) (Fig. 3). Although the weighted median method did not yield a significant causal relationship between serum TAG (51:4) levels and PD risk (OR = 0·76, weighted median, *P*= 0·079), the results indicate agreement with the other methods.

### Association of serum TAG (51:4) levels with circulating immune cell counts and circulating inflammatory protein levels

**Figure 4.**
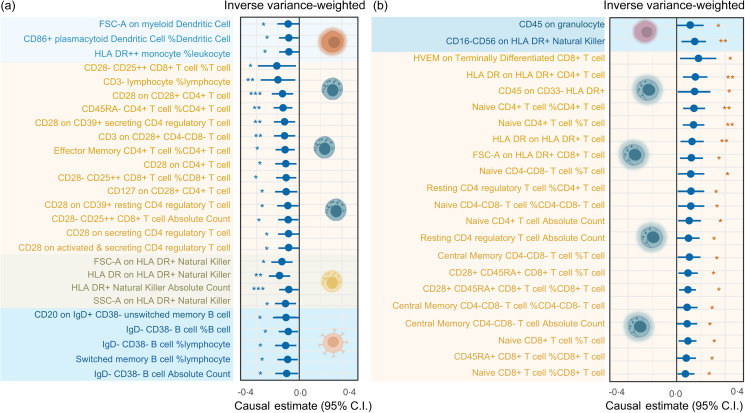
Forest plot from MR analyses testing the effect of serum TAG (51:4) levels on various circulating immune cell counts. The results of the five MR analysis approaches are presented as causal estimates and 95 % CI. MR, Mendelian randomisation; IVW, inverse variance-weighted. *P* values less than 0·05 are indicated by *, 0·01 and 0·001 by **, *** and *, respectively.

Additionally, we discovered that there may be a causal relationship between serum TAG (51:4) levels and the counts of various subtypes of circulating immune cells. Lower T lymphocyte counts from fourteen different subtypes were linked to elevated blood TAG (51:4) levels (Fig. 4). Among them, the relative count of immature CD28- CD25++ CD8 + T cells (immature CD28- CD25++ CD8 + T cells %) decreased most significantly (with 1 sd genetically instrumented higher serum TAG (51:4) level leading to a 21 per cent (95 % CI −0·38, −0·03) lower immature CD28- CD25++ CD8 + T cells %, *P*= 0·021). Additionally, our findings indicated that increased serum TAG (51:4) levels were associated with reductions in the count of five subtypes of B lymphocyte (CD20 on IgD + CD38- unswitched memory B cells, IgD- CD38- B cell %, IgD- CD38- B cell % lymphocytes, IgD- CD38- B cell absolute counts and switched memory B cell % lymphocytes), two subtypes of dendritic cells (FSC-A on myeloid dendritic cells and CD86+ plasmacytoid dendritic cells %), four subtypes of natural killer (FSC-A on HLA DR+ natural killer, HLA DR on HLA DR+ natural killer, HLA DR+ natural killer absolute count and SSC-A on HLA DR+ natural killer) and the HLA DR++ monocyte %leucocyte (Fig. 4(a)).

The IVW methods also demonstrated a potential causal relationship between increased serum TAG (51:4) levels and an increase in the counts of 20 T lymphocyte cell subtypes. Among them, the most significant increase was found in the Herpes Virus Entry Mediator (HVEM) on terminally differentiated CD8 + T cell count (with 1 sd genetically instrumented higher serum TAG (51:4) levels leading to an 18 % (95 % CI 0·03, 0·33) higher HVEM on terminally differentiated CD8 + T cells, *P*= 0·020) (Fig. 3(b)). In addition to T lymphocytes, our MR results also showed a potential causal relationship between increased serum TAG (51:4) levels and an increase in the count of CD16-CD56 on HLA-DR + natural killer and CD45 on granulocyte cells (Fig. 4(b)).

**Figure 5.**
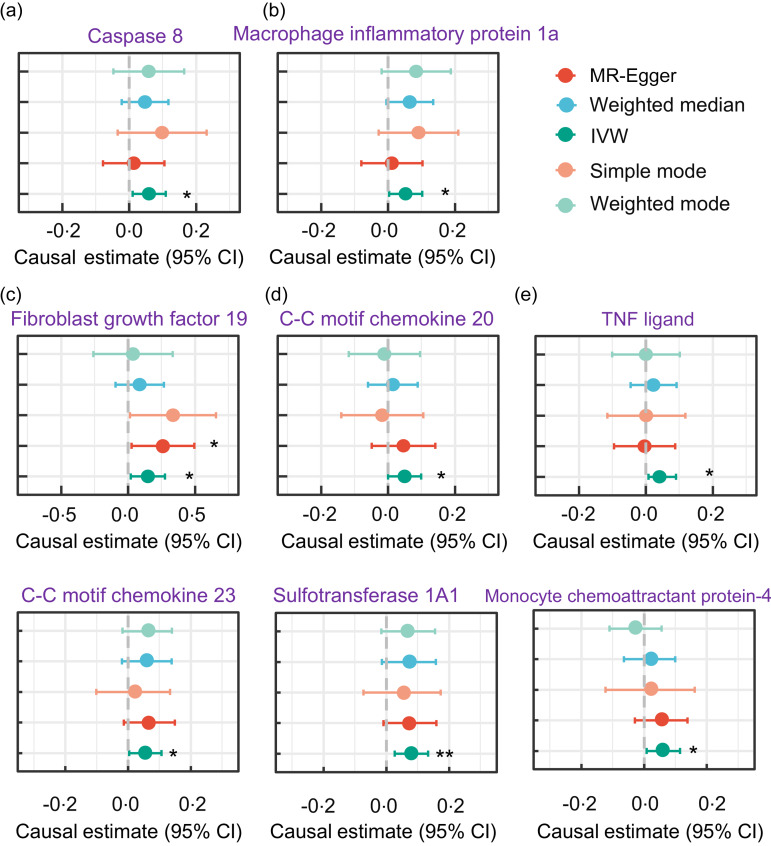
Forest plot MR analyses testing the effect of serum TAG (51:4) levels on various circulating inflammatory proteins levels. The results of the five MR analysis methods were presented as causal estimates and 95 % CI. MR, Mendelian randomisation. * indicates a *P*-value less than 0·05, ** denotes a *P*-value less than 0·01 and *** denotes a *P*-value less than 0·001.

We also examined the association between serum TAG (51:4) levels and circulating inflammatory proteins. Notably, we found a positive causal relationship between increased serum TAG (51:4) levels and levels of eight circulating inflammatory proteins (with 1 sd genetically instrumented higher serum TAG (51:4) levels leading to a 6 % (95 % CI 1·01, 1·11) higher caspase 8 levels, *P*= 0·013; 6 % (95 % CI 1·01, 1·11) higher C-C motif chemokine 20 levels, *P*= 0·024; 8 % (95 % CI 1·02, 1·14) higher fibroblast growth factor 19 levels, *P*= 0·006; 6 % (95 % CI 1·1, 1·11) higher macrophage inflammatory protein 1a levels, *P*= 0·017; and 5 % (95 % CI 1·01, 1·10) TNF ligand superfamily member 12, *P*= 0·049) (Fig. 5).

### Association of circulating immune cell counts with Parkinson’s disease

**Figure 6.**
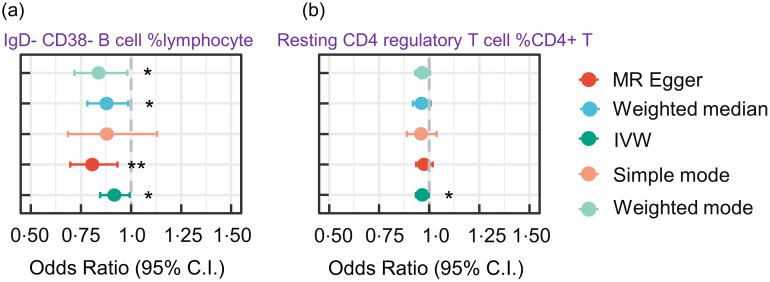
Forest plot for MR analyses testing the effect of circulating immune cells on the risk of PD. The results of the five MR analysis methods are presented as OR and 95 % CI. * indicates a *P*-value less than 0·05, ** denotes a *P*-value less than 0·01 and *** denotes a *P*-value less than 0·001. MR, Mendelian randomisation; PD, Parkinson’s disease; IVW, inverse variance-weighted.

Our MR analysis provided robust evidence supporting a causal relationship between the number of circulating immune cells and increased risk of PD. Specifically, increased IgD-CD38-B lymphocyte counts were associated with a reduction in the risk of PD (IVW OR, 0·91 (95 % CI 0·85, 0·99), *P*= 0·006). This suggests that the risk of PD is decreased by 9 % with 1 % genetically instrumented higher circulating IgD-CD38-B cell % lymphocyte count. Additionally, we found that increased resting CD4 regulatory T cell %CD4 + T cell counts were associated with a reduction in the risk of PD (with a 1 % genetically instrumented higher resting CD4 regulatory T cell %CD4 + T cell leading to a 4 % (95 % CI 0·93, 0·99) lower risk of PD, *P*= 0·038) (Fig. 6).

### Proportion of the association between serum TAG (51:4) levels and Parkinson’s disease mediated by circulating immune cell counts

The effect of serum TAG (51:4) levels was partially mediated by the IgD-CD38-B cell for all-cause PD (proportion of circulating IgD-CD38-B cell mediated: 6·9 %; CI 2·1 %, 16 %; *P*= 0·024). Additionally, we found that the mediating effect of resting CD4 regulatory T cells in association with serum TAG (51:4) levels and PD was not significant (proportion of circulating resting CD4 regulatory T cell mediated: 3·7 %; CI −6·5 %, 14 %; *P*= 0·336) (Table 1). Interestingly, none of the ninety-one circulating inflammatory proteins, except circulating immune cells, mediated the relationship between serum TAG (51:4) levels and PD risk.

**Table 1.** The estimated proportions of the association between serum TAG (51:4) levels and the risk of PD accounted for by circulating immune cells

Mediators	Methods	% Proportion mediated	95 % CI	*P*
IgD-CD38-B cell % lymphocyte	IVW	6·9	2·1, 16	0·024
Resting CD4 regulatory T cell %CD4 + T cell	IVW	5·7	6·8, 18	0·475

PD, Parkinson’s disease; IVW, inverse variance weighted.

### Sensitivity analysis

Online Supplementary Tables S1–S8 summarise the findings of our sensitivity analyses, which included pleiotropy and heterogeneity tests. Notably, we found no evidence of horizontal pleiotropy for circulating inflammatory proteins, immune cells or serum TAG (51:4) levels (*P*> 0·05). Additionally, all analytical findings showed concordance between the IVW and MR-Egger results, with no indication of heterogeneity (*P*> 0·05).

## Discussion

According to genetic variant analysis, lipid and lipid transport, autophagic-lysosomal pathways, and inflammatory pathways are now known to carry a substantial and influential genetic risk for age-dependent neurological illnesses^(17,57,58)^. However, the mechanisms through which these molecular interactions contribute to the pathology of many age-related neurodegenerative illnesses, including dementia, Alzheimer’s disease and PD, remain poorly understood. We used GWAS summary data to evaluate for a causal relationship between plasma lipidomes and PD, related primarily to serum TAG (51:4) levels and PD. We also attempted to clarify a possible role for mediation through circulating immune cells but not circulating inflammatory proteins.

Several observational studies^(59–61)^ have examined the relationship between lipid metabolism and PD onset. However, the specific types of lipid metabolism disturbances associated with the risk of PD remain unclear. In the present study, which included 179 lipid species in 7174 adult Finnish subjects, we employed MR analysis to investigate the potential association between plasma lipidomes and PD risk. The MR findings demonstrated a causative relationship between higher serum TAG (51:4) levels and a reduced risk of PD. Consistent with our results, a meta-analysis showed that higher levels of total serum TAG and cholesterol were associated with reduced PD risk or were higher in control group compared with the PD group^(62)^. Similarly, several clinical observational studies have reported lower serum TAG levels in patients with PD compared with healthy individuals^(63–65)^. Further, several clinical studies have also demonstrated that higher serum levels of TAG confer protective benefits against PD^(66,67)^. Additionally, the findings of animal experiments revealed that mice overexpressing *α*-syn A53T and other PD models have considerably lower intracellular TAG levels^(65,68)^. However, Zhang *et al.* discovered a correlation between worse motor performance and higher serum TAG levels in patients with PD^(35)^. We inferred that this may be due to confounding effects in observational studies, which is efficiently eliminated by MR analysis.

Evidence suggests that a variety of lipid species, including sterols, fatty acids and their metabolites, complex lipids (such as glycerophospholipids and sphingolipids) and lipoproteins, have immunomodulatory, and pro- and anti-inflammatory properties^(36)^. Thus, lipid metabolism is crucial in controlling inflammation in acute and chronic illnesses, including Alzheimer’s disease and PD. Since lipids possess pro- and anti-inflammatory properties and lipoprotein profiles and composition affect immunomodulatory pathways, we explored the role that phenotypes associated with 731 different immune cells and ninety-one distinct circulating inflammatory protein phenotypes play as mediators in the relationship between the plasma lipidome and PD. Interestingly, we found no evidence supporting the mediating role of circulating inflammatory proteins; instead, we identified a potential mediating role for circulating immune cells. More precisely, our results showed that higher percentages of resting CD4 regulatory T cells (%CD4 + T cells) and IgD-CD38-B cells (% lymphocytes) were associated with a lower risk of PD (IVW OR, 0·91 (95 % CI 0·85, 0·99), *P*= 0·033 and 0·96 (95 % CI 0·93, 0·99), *P*= 0·038, respectively). Consistent with our findings, Stienstra *et al.* discovered that lowering adipose TAG lipase-mediated lipolysis led to a reduction in PG-E2 and IL-6 production, which in turn attenuated the inflammatory response in macrophages following *ex vivo* and *in vitro* activation^(26)^. Similarly, Fülöp T and colleagues discovered that changes in plasma membrane characteristics are linked to a suppressive effect on peripheral T-cell CD28-dependent activation caused by selective increase in intravascular lipolysis and plasma linoleic acid content^(69)^. Additionally, mediating roles of IgD + CD38dim%B and CD25 ++ CD8br in the causal link between TAG (51:4) and onset of hypotension have been validated in recent MR research^(70)^. Overall, our results suggest that circulating immune cells play a major mediating role in the relationship between serum TAG (51:4) levels and PD risk.

As components of the adaptive immune system, B lymphocytes perform a range of functions and intricately interact with other pathways of the innate and adaptive immune systems. Recent studies have indicated that immune cells, including B lymphocytes, may engage in intricate interactions with the central nervous system through the meningeal lymphatic system^(71,72)^. Other studies have identified channels in the skull bone marrow that permit the egress of calvarial immune cells, including B lymphocytes^(73,74)^. These findings imply that B lymphocytes potentially contribute significantly to the inflammatory process in PD, both centrally and peripherally, indicating that they may be a pertinent target for disease-modifying treatments. Notably, it is now well accepted that B cell counts are reduced in PD^(75–78)^, which is consistent with our results. Importantly, postmortem research has shown that IgG binds to approximately 30 % of neurons in the substantia nigra^(79)^. We found a causal relationship between increased serum TAG (51:4) levels and a reduction in the percentage of IgD-CD38-B lymphocytes. More significantly, a causal association was present between a lower percentage of IgD-CD38-B lymphocytes and reduced risk of PD. These findings indicate that IgD-CD38-B cell lymphocytes mediate the effects of serum TAG (51:4) levels on PD.

In addition to B lymphocytes, several animal models have demonstrated the important role of Treg cells in preventing neurodegeneration in PD. A decrease in IFN-g-producing type 1 helper T cells was correlated with an increase in CD41CD251 cells in lymphoid tissues in the 1-methyl-4-phenyl-1,2,3,6-tetrahydropyridine model^(80)^. Similarly, a more robust microglial response and greater loss of dopaminergic neurons in the MPTP model are linked to considerably lower numbers of CD41CD251 cells observed in aquaporin-4 knockout mice when compared with wild-type mice^(81)^. In the same model, systemically increasing the number of Treg cells by injecting Treg-expanding bee venom phospholipase A2 or adoptive transfer of CD3-activated Treg cells prevented the degeneration of dopaminergic neurons in the substantia nigra and reduced microglial activation and CD41 cell infiltration^(82)^. Furthermore, growing evidence suggests that Treg cell malfunction may play a role in the pathophysiology of PD. PTEN-induced kinase 1 (PINK1) mutations are associated with familial early-onset parkinsonism, and Treg cell formation and function are impaired in PINK1 knockout mice^(83)^. Although Treg cells have been reported to have a greater suppressive capacity in patients with PD^(84)^, other studies have revealed that this condition reduces serum Treg cell counts^(85)^ and affects their ability to suppress effector T cell function *in vitro*^(86)^. We found that there was a causal relationship between higher serum TAG (51:4) levels and an increase in the proportion of resting CD4 regulatory T cells %CD4 + T cells. More importantly, a causal relationship was observed between a lower risk of PD and a greater ratio of resting CD4 regulatory T cells to CD4 + T cells, which was revealed as another mediator in the relationship between plasma lipidome exposure and the risk of PD. Consequently, we speculated that the effect of serum TAG (51:4) levels was partially mediated by resting CD4 regulatory T cells and CD4 + T cells.

Human cross-sectional and cohort studies have highlighted that high total fat intake can increase the risk of allergy and allergic diseases^(87)^. High total fat intake, especially saturated fat, often leads to increased TAG levels in the blood, which may exacerbate neuroinflammation and neurodegeneration in PD^(88–90)^. Notably, the effect of dysregulated lipid metabolism on the risk of PD, specifically with regard to TAG, remains unclear. Our results are important, in part, as they offer a mechanistic explanation for the association between blood TAG (51:4) levels and development of PD, which has been observed and demonstrated in other studies. Several studies have indicated a strong relationship between oxidative stress and chronic inflammation caused by endogenous danger signalling molecules, as well as the onset and progression of PD^(91–93)^. Notably, nutritional studies have shown that TAG derived from red sorghum have potent antioxidant, anti-inflammatory and radical scavenging properties^(94)^. Furthermore, Deng *et al.* verified that dietary consumption of medium- and long-chain TAG considerably enhanced lipid metabolism and decreased inflammation in obese rats fed a high-fat diet^(95)^. Additionally, Campos *et al.* found esterification of arachidonic acid into TAGs in Fe-overloaded neurons, and an increase in TAG levels and appearance of lipid droplets suggested that dopaminergic neurons respond to oxidative injury by storing peroxidable fatty acids in the TAG pool, thus avoiding the persistence of PUFA in the phospholipid fraction^(96)^. These results imply that the anti-inflammatory and antioxidant properties of TAG (51:4) may be the underlying mechanisms by which it protects against PD.

In addition to modulating immune responses, TAG play a vital role in improving lipid metabolism, particularly within glial cells, which may be crucial for maintaining neuronal health and homoeostasis^(97)^. Enhanced lipid metabolism helps mitigate disruptions that could otherwise lead to neurodegeneration^(98)^. Studies have suggested that higher TAG levels are often associated with neuroprotection in the context of PD, potentially reducing the risk of developing the condition and promoting better outcomes in affected individuals^(99)^. Additionally, TAG are integral to energy metabolism in the brain and essential for sustaining neuronal function. An adequate energy supply is critical for maintaining neuronal integrity, and disruptions in energy metabolism can lead to neurodegenerative changes^(97)^. By supporting energy metabolism, TAG contribute to overall neuronal health and may prevent the progression of neurodegenerative diseases, such as PD^(100)^. Interestingly, autophagy activation, a critical cellular degradation pathway, is another mechanism by which TAG exhibit protective effects^(101)^. Enhanced autophagic activity linked to TAG may promote clearance of misfolded proteins and damaged organelles, which are characteristic of neurodegenerative diseases, such as PD^(102)^. This process could be particularly effective in reducing protein aggregation, such as that of *α*-syn, which has been implicated in the pathology of PD. Additionally, TAG stored in lipid droplets may serve as buffers against toxins and oxidative stress that contribute to PD. For instance, synucleins, which are proteins linked to PD, are known to bind TAG and protect them from degradation^(103)^, thereby potentially shielding cells against PD-related damage. In summary, TAG, a type of lipid, may offer protection against PD through several mechanisms, although the exact process is not fully understood.

### Limitations

Understanding the estimates obtained by MR can be challenging, as MR depends on assumptions that might not be testable, such as exclusion criteria, especially in cases involving unmeasured or unknown potential confounding factors. For example, MR estimates may be confounded by horizontal pleiotropy, a phenomenon in which genetic variants are independently associated with traits other than those studied. Sensitivity analyses that are more resilient to these types of pleiotropy can be employed to assess the susceptibility of MR estimates to horizontal pleiotropy. In this study, the MR-Egger and weighted median approaches were utilised for this purpose. Additionally, allele frequencies and illness risk range between populations of various genetic ancestries might introduce genetic confounders into an MR study and potentially lead to false causal estimations. The GWAS approaches used in this investigation to identify associations between circulating immune cell counts and PD considered population stratification and cryptic relatedness should minimise the chances of these confounding factors.

The present study found evidence supporting a potential causal relationship between the reduction in PD risk and higher serum TAG (51:4) levels and clarified the possible role of circulating immune cells (including IgD-CD38-B cell lymphocytes and resting CD4 regulatory T cell %CD4 + T cells), but not circulating inflammatory proteins. The molecular mechanisms underlying this association may involve pathways associated with inflammation; however, they can also operate independently of classical regulatory mechanisms.

### Suggestions for future work

Some studies have found that therapeutic strategies aimed at lipid metabolism in PD can significantly alleviate disease progression by reducing the aggregation of *α*-syn^(104)^. For instance, targeting specific enzymes involved in lipid metabolism, such as LIPE, may decrease *α*-syn inclusions and improve neuronal health^(105)^. Enhanced fatty acid metabolism can also facilitate energy production and reduce oxidative stress, which are detrimental to dopaminergic neurons. Immune modulation is another promising approach for therapeutic intervention in PD. It is generally accepted that chronic neuroinflammation is a key contributor to dopaminergic neuron degeneration, and targeting inflammatory pathways may protect neuronal integrity^(106)^. Findings based on the present study, thus, combining lipid metabolism modulation with immune modulation may be a potent therapeutic strategy. For example, interventions that reduce neuroinflammation by targeting specific enzymes that regulate lipid metabolism in neurons may synergistically improve patient outcomes. A multifaceted approach may facilitate the reduction of toxic protein aggregates, while simultaneously supporting overall brain health.

## Supporting information

Jing et al. supplementary material Jing et al. supplementary material

## Supplementary material

For supplementary material/s referred to in this article, please visit https://doi.org/10.1017/S0007114524002137

## References

[1] Van Den Eeden SK , Tanner CM , Bernstein AL , et al. (2003) Incidence of Parkinson’s disease: variation by age, gender, and race/ethnicity. Am J Epidemiol 157, 1015–1022. 12777365 10.1093/aje/kwg068

[2] Lampropoulos IC , Malli F , Sinani O , et al. (2022) Worldwide trends in mortality related to Parkinson’s disease in the period of 1994–2019: analysis of vital registration data from the WHO mortality database. Front Neurol 13, 956440. 36267881 10.3389/fneur.2022.956440 PMC9576872

[3] Tysnes OB & Storstein A (2017) Epidemiology of Parkinson’s disease. J Neural Transm (Vienna) 124, 901–905. 28150045 10.1007/s00702-017-1686-y

[4] Ou Z , Pan J , Tang S , et al. (2021) Global trends in the incidence, prevalence, and years lived with disability of Parkinson’s disease in 204 countries/territories from 1990 to 2019. Front Public Health 9, 776847. 34950630 10.3389/fpubh.2021.776847 PMC8688697

[5] Dorsey ER , Elbaz A , Nichols E , et al. (2018) Global, regional, and national burden of Parkinson’s disease, 1990–2016: a systematic analysis for the Global Burden of Disease Study 2016. Lancet Neurol 17, 939–953. 30287051 10.1016/S1474-4422(18)30295-3 PMC6191528

[6] Baizabal-Carvallo JF & Jankovic J (2016) Parkinsonism, movement disorders and genetics in frontotemporal dementia. Nat Rev Neurol 12, 175–185. 26891767 10.1038/nrneurol.2016.14

[7] Schonhoff AM , Williams GP , Wallen ZD , et al. (2020) Innate and adaptive immune responses in Parkinson’s disease. Prog Brain Res 252, 169–216. 32247364 10.1016/bs.pbr.2019.10.006 PMC7185735

[8] Wallings RL , Humble SW , Ward ME , et al. (2019) Lysosomal dysfunction at the centre of Parkinson’s disease and frontotemporal dementia/amyotrophic lateral sclerosis. Trends Neurosci 42, 899–912. 31704179 10.1016/j.tins.2019.10.002 PMC6931156

[9] Juárez-Flores DL , Ezquerra M , Gonzàlez-Casacuberta Ï , et al. (2020) Disrupted mitochondrial and metabolic plasticity underlie comorbidity between age-related and degenerative disorders as Parkinson disease and type 2 diabetes mellitus. Antioxidants (Basel) 9, 1063. 33143119 10.3390/antiox9111063 PMC7693963

[10] Robea MA , Balmus IM , Ciobica A , et al. (2020) Parkinson’s disease-induced Zebrafish models: focussing on oxidative stress implications and sleep processes. Oxid Med Cell Longev 2020, 1370837. 32908622 10.1155/2020/1370837 PMC7450359

[11] Raza C , Anjum R & Shakeel NUA (2019) Parkinson’s disease: mechanisms, translational models and management strategies. Life Sci 226, 77–90. 30980848 10.1016/j.lfs.2019.03.057

[12] Grayson M (2016) Parkinson’s disease. Nature 538, S1. 27783582 10.1038/538S1a

[13] Mori A , Imai Y & Hattori N (2020) Lipids: key players that modulate *α*-synuclein toxicity and neurodegeneration in Parkinson’s disease. Int J Mol Sci 21, 3301. 32392751 10.3390/ijms21093301 PMC7247581

[14] Neumann J , Bras J , Deas E , et al. (2009) Glucocerebrosidase mutations in clinical and pathologically proven Parkinson’s disease. Brain 132, 1783–1794. 19286695 10.1093/brain/awp044 PMC2702833

[15] Sidransky E , Nalls MA , Aasly JO , et al. (2009) Multicenter analysis of glucocerebrosidase mutations in Parkinson’s disease. N Engl J Med 361, 1651–1661. 19846850 10.1056/NEJMoa0901281 PMC2856322

[16] Gan-Or Z , Ozelius LJ , Bar-Shira A , et al. (2013) The p.L302P mutation in the lysosomal enzyme gene SMPD1 is a risk factor for Parkinson disease. Neurology 80, 1606–1610. 23535491 10.1212/WNL.0b013e31828f180e PMC3662322

[17] Chang D , Nalls MA , Hallgrímsdóttir IB , et al. (2017) A meta-analysis of genome-wide association studies identifies 17 new Parkinson’s disease risk loci. Nat Genet 49, 1511–1516. 28892059 10.1038/ng.3955 PMC5812477

[18] Robak LA , Jansen IE , van Rooij J , et al. (2017) Excessive burden of lysosomal storage disorder gene variants in Parkinson’s disease. Brain 140, 3191–3203. 29140481 10.1093/brain/awx285 PMC5841393

[19] Simón-Sánchez J , Schulte C , Bras JM , et al. (2009) Genome-wide association study reveals genetic risk underlying Parkinson’s disease. Nat Genet 41, 1308–1312. 19915575 10.1038/ng.487 PMC2787725

[20] Nalls MA , Pankratz N , Lill CM , et al. (2014) Large-scale meta-analysis of genome-wide association data identifies six new risk loci for Parkinson’s disease. Nat Genet 46, 989–993. 25064009 10.1038/ng.3043 PMC4146673

[21] Goldschmidt HL , Tu-Sekine B , Volk L , et al. (2016) DGKθ Catalytic activity is required for efficient recycling of presynaptic vesicles at excitatory synapses. Cell Rep 14, 200–207. 26748701 10.1016/j.celrep.2015.12.022 PMC4715728

[22] Puchkov D & Haucke V (2013) Greasing the synaptic vesicle cycle by membrane lipids. Trends Cell Biol 23, 493–503. 23756446 10.1016/j.tcb.2013.05.002

[23] Do CB , Tung JY , Dorfman E , et al. (2011) Web-based genome-wide association study identifies two novel loci and a substantial genetic component for Parkinson’s disease. PLoS Genet 7, e1002141. 21738487 10.1371/journal.pgen.1002141 PMC3121750

[24] Lee JH , Phelan P , Shin M , et al. (2018) SREBP-1a-stimulated lipid synthesis is required for macrophage phagocytosis downstream of TLR4-directed mTORC1. Proc Natl Acad Sci USA 115, E12228–e34. 30530672 10.1073/pnas.1813458115 PMC6310840

[25] Shahmoradian SH , Lewis AJ , Genoud C , et al. (2019) Lewy pathology in Parkinson’s disease consists of crowded organelles and lipid membranes. Nat Neurosci 22, 1099–1109. 31235907 10.1038/s41593-019-0423-2

[26] van Dierendonck X , Vrieling F , Smeehuijzen L , et al. (2022) Triglyceride breakdown from lipid droplets regulates the inflammatory response in macrophages. Proc Natl Acad Sci USA 119, e2114739119. 35302892 10.1073/pnas.2114739119 PMC8944848

[27] McLelland GL , Lopez-Osias M , Verzijl CRC , et al. (2023) Identification of an alternative triglyceride biosynthesis pathway. Nature 621, 171–178. 37648867 10.1038/s41586-023-06497-4 PMC10482677

[28] Xu C & Shanklin J (2016) Triacylglycerol metabolism, function, and accumulation in plant vegetative tissues. Annu Rev Plant Biol 67, 179–206. 26845499 10.1146/annurev-arplant-043015-111641

[29] Shulman GI (2014) Ectopic fat in insulin resistance, dyslipidemia, and cardiometabolic disease. N Engl J Med 371, 2237–2238. 25470706 10.1056/NEJMc1412427

[30] Quehenberger O & Dennis EA (2011) The human plasma lipidome. N Engl J Med 365, 1812–1823. 22070478 10.1056/NEJMra1104901 PMC3412394

[31] Cases S , Smith SJ , Zheng YW , et al. (1998) Identification of a gene encoding an acyl CoA:diacylglycerol acyltransferase, a key enzyme in triacylglycerol synthesis. Proc Natl Acad Sci USA 95, 13018–13023. 9789033 10.1073/pnas.95.22.13018 PMC23692

[32] Power MC , Rawlings A , Sharrett AR , et al. (2018) Association of midlife lipids with 20-year cognitive change: a cohort study. Alzheimers Dement 14, 167–177. 28916238 10.1016/j.jalz.2017.07.757 PMC5803364

[33] Kalmijn S , Foley D , White L , et al. (2000) Metabolic cardiovascular syndrome and risk of dementia in Japanese-American elderly men. The Honolulu-Asia aging study. Arterioscler Thromb Vasc Biol 20, 2255–2260. 11031212 10.1161/01.atv.20.10.2255

[34] Huang X , Ng SY , Chia NS , et al. (2018) Higher serum triglyceride levels are associated with Parkinson’s disease mild cognitive impairment. Mov Disord 33, 1970–1971. 30345542 10.1002/mds.27521

[35] Zhang M , Chen H , Liu G , et al. (2022) Lower serum triglyceride levels linked to more severe motor performance in Parkinson’s disease. Neurol Sci 43, 5343–5353. 35608738 10.1007/s10072-022-06113-9 PMC9385747

[36] Andersen CJ (2022) Lipid metabolism in inflammation and immune function. Nutrients 14, 1414. 35406026 10.3390/nu14071414 PMC9002396

[37] Araújo B , Caridade-Silva R , Soares-Guedes C , et al. (2022) Neuroinflammation and Parkinson’s disease-from neurodegeneration to therapeutic opportunities. Cells 11, 2908. 36139483 10.3390/cells11182908 PMC9497016

[38] Ottensmann L , Tabassum R , Ruotsalainen SE , et al. (2023) Genome-wide association analysis of plasma lipidome identifies 495 genetic associations. Nat Commun 14, 6934. 37907536 10.1038/s41467-023-42532-8 PMC10618167

[39] Orrù V , Steri M , Sidore C , et al. (2020) Complex genetic signatures in immune cells underlie autoimmunity and inform therapy. Nat Genet 52, 1036–1045. 32929287 10.1038/s41588-020-0684-4 PMC8517961

[40] Zhao JH , Stacey D , Eriksson N , et al. (2023) Genetics of circulating inflammatory proteins identifies drivers of immune-mediated disease risk and therapeutic targets. Nat Immunol 24, 1540–1551. 37563310 10.1038/s41590-023-01588-w PMC10457199

[41] Sakaue S , Kanai M , Tanigawa Y , et al. (2021) A cross-population atlas of genetic associations for 220 human phenotypes. Nat Genet 53, 1415–1424. 34594039 10.1038/s41588-021-00931-x PMC12208603

[42] Chang CC , Chow CC , Tellier LC , et al. (2015) Second-generation PLINK: rising to the challenge of larger and richer datasets. GigaScience 4, 7. 25722852 10.1186/s13742-015-0047-8 PMC4342193

[43] van der Graaf A , Zorro MM , Claringbould A , et al. (2020) Systematic prioritization of candidate genes in disease loci identifies TRAFD1 as a master regulator of IFN*γ* signaling in celiac disease. Front Genet 11, 562434. 33569077 10.3389/fgene.2020.562434 PMC7868554

[44] Hemani G , Zheng J , Elsworth B , et al. (2018) The MR-Base platform supports systematic causal inference across the human phenome. Elife 7, e34408. 29846171 10.7554/eLife.34408 PMC5976434

[45] Bowden J , Davey Smith G , Haycock PC , et al. (2016) Consistent estimation in Mendelian randomization with some invalid instruments using a weighted median estimator. Genet Epidemiol 40, 304–314. 27061298 10.1002/gepi.21965 PMC4849733

[46] Bowden J , Davey Smith G & Burgess S (2015) Mendelian randomization with invalid instruments: effect estimation and bias detection through Egger regression. Int J Epidemiol 44, 512–525. 26050253 10.1093/ije/dyv080 PMC4469799

[47] Slob EAW & Burgess S (2020) A comparison of robust Mendelian randomization methods using summary data. Genet Epidemiol 44, 313–329. 32249995 10.1002/gepi.22295 PMC7317850

[48] Davey Smith G & Hemani G (2014) Mendelian randomization: genetic anchors for causal inference in epidemiological studies. Hum Mol Genet 23, R89–R98. 25064373 10.1093/hmg/ddu328 PMC4170722

[49] Sanderson E , Spiller W & Bowden J (2021) Testing and correcting for weak and pleiotropic instruments in two-sample multivariable Mendelian randomization. Stat Med 40, 5434–5452. 34338327 10.1002/sim.9133 PMC9479726

[50] Bowden J , Hemani G & Davey Smith G (2018) Invited commentary: detecting individual and global horizontal pleiotropy in Mendelian randomization-a job for the humble heterogeneity statistic? Am J Epidemiol 187, 2681–2685. 30188969 10.1093/aje/kwy185 PMC6269239

[51] Verbanck M , Chen CY , Neale B , et al. (2018) Detection of widespread horizontal pleiotropy in causal relationships inferred from Mendelian randomization between complex traits and diseases. Nat Genet 50, 693–698. 29686387 10.1038/s41588-018-0099-7 PMC6083837

[52] Sanderson E , Glymour MM , Holmes MV , et al. (2022) Mendelian randomization. Nat Rev Methods Primers 2, 6. 37325194 10.1038/s43586-021-00092-5 PMC7614635

[53] Burgess S , Davies NM & Thompson SG (2016) Bias due to participant overlap in two-sample Mendelian randomization. Genet Epidemiol 40, 597–608. 27625185 10.1002/gepi.21998 PMC5082560

[54] Mounier N & Kutalik Z (2023) Bias correction for inverse variance weighting Mendelian randomization. Genet Epidemiol 47, 314–331. 37036286 10.1002/gepi.22522

[55] Hu X , Zhao J , Lin Z , et al. (2022) Mendelian randomization for causal inference accounting for pleiotropy and sample structure using genome-wide summary statistics. Proc Natl Acad Sci USA 119, e2106858119. 35787050 10.1073/pnas.2106858119 PMC9282238

[56] Zheng J , Erzurumluoglu AM , Elsworth BL , et al. (2017) LD hub: a centralized database and web interface to perform LD score regression that maximizes the potential of summary level GWAS data for SNP heritability and genetic correlation analysis. Bioinf 33, 272–279. 10.1093/bioinformatics/btw613 PMC5542030 27663502

[57] Shi H , Belbin O , Medway C , et al. (2012) Genetic variants influencing human aging from late-onset Alzheimer’s disease (LOAD) genome-wide association studies (GWAS). Neurobiol Aging 33, 1849.e5–18. 10.1016/j.neurobiolaging.2012.02.014 PMC4120742 22445811

[58] Klemann C , Martens GJM , Sharma M , et al. (2017) Integrated molecular landscape of Parkinson’s disease. NPJ Parkinsons Dis 3, 14. 28649614 10.1038/s41531-017-0015-3 PMC5460267

[59] Coulombe K , Kerdiles O , Tremblay C , et al. (2018) Impact of DHA intake in a mouse model of synucleinopathy. Exp Neurol 301, 39–49. 29229294 10.1016/j.expneurol.2017.12.002

[60] Wang Q , Liu Y & Zhou J (2015) Neuroinflammation in Parkinson’s disease and its potential as therapeutic target. Transl Neurodegener 4, 19. 26464797 10.1186/s40035-015-0042-0 PMC4603346

[61] Kubo S , Nemani VM , Chalkley RJ , et al. (2005) A combinatorial code for the interaction of *α* -synuclein with membranes. J Biol Chem 280, 31664–31672. 10.1074/jbc.M504894200 16020543

[62] Fu X , Wang Y , He X , et al. (2020) A systematic review and meta-analysis of serum cholesterol and triglyceride levels in patients with Parkinson’s disease. Lipids Health Dis 19, 97. 32430016 10.1186/s12944-020-01284-w PMC7236933

[63] Scigliano G , Musicco M , Soliveri P , et al. (2006) Reduced risk factors for vascular disorders in Parkinson disease patients: a case-control study. Stroke 37, 1184–1188. 16574924 10.1161/01.STR.0000217384.03237.9c

[64] Guo X , Song W , Chen K , et al. (2015) The serum lipid profile of Parkinson’s disease patients: a study from China. Int J Neurosci 125, 838–844. 25340257 10.3109/00207454.2014.979288

[65] Meng X , Zheng R , Zhang Y , et al. (2015) An activated sympathetic nervous system affects white adipocyte differentiation and lipolysis in a rat model of Parkinson’s disease. J Neurosci Res 93, 350–360. 25257318 10.1002/jnr.23488

[66] Sääksjärvi K , Knekt P , Männistö S , et al. (2015) Prospective study on the components of metabolic syndrome and the incidence of Parkinson’s disease. Parkinsonism Relat Disord 21, 1148–1155. 26228080 10.1016/j.parkreldis.2015.07.017

[67] Vikdahl M , Bäckman L , Johansson I , et al. (2015) Cardiovascular risk factors and the risk of Parkinson’s disease. Eur J Clin Nutr 69, 729–733. 25514902 10.1038/ejcn.2014.259

[68] Guerreiro PS , Coelho JE , Sousa-Lima I , et al. (2017) Mutant A53T *α*-synuclein improves rotarod performance before motor deficits and affects metabolic pathways. Neuromolecular Med 19, 113–121. 27535567 10.1007/s12017-016-8435-5

[69] Larbi A , Grenier A , Frisch F , et al. (2005) Acute *in vivo* elevation of intravascular triacylglycerol lipolysis impairs peripheral T cell activation in humans. Am J Clin Nutr 82, 949–956. 16280424 10.1093/ajcn/82.5.949

[70] Lin W , Han N , Hong Q , et al. (2024) Role of immune cells in mediating the effect of triacylglycerol (50:2) on hypotension, 21 April 2024, PREPRINT (Version 1) available at Research Square [10.21203/rs.3.rs-4232038/v1].

[71] Louveau A , Herz J , Alme MN , et al. (2018) CNS lymphatic drainage and neuroinflammation are regulated by meningeal lymphatic vasculature. Nat Neurosci 21, 1380–1391. 30224810 10.1038/s41593-018-0227-9 PMC6214619

[72] Zou W , Pu T , Feng W , et al. (2019) Blocking meningeal lymphatic drainage aggravates Parkinson’s disease-like pathology in mice overexpressing mutated *α*-synuclein. Transl Neurodegener 8, 7. 30867902 10.1186/s40035-019-0147-y PMC6396507

[73] Herisson F , Frodermann V , Courties G , et al. (2018) Direct vascular channels connect skull bone marrow and the brain surface enabling myeloid cell migration. Nat Neurosci 21, 1209–1217. 30150661 10.1038/s41593-018-0213-2 PMC6148759

[74] Kolabas ZI , Kuemmerle LB , Perneczky R , et al. (2021) Multi-omics and 3D-imaging reveal bone heterogeneity and unique calvaria cells in neuroinflammation. bioRxiv 2021.12.24.473988.

[75] Gruden MA , Sewell RD , Yanamandra K , et al. (2011) Immunoprotection against toxic biomarkers is retained during Parkinson’s disease progression. J Neuroimmunol 233, 221–227. 21239064 10.1016/j.jneuroim.2010.12.001

[76] Kedmi M , Bar-Shira A , Gurevich T , et al. (2011) Decreased expression of B cell related genes in leukocytes of women with Parkinson’s disease. Mol Neurodegener 6, 66. 21943286 10.1186/1750-1326-6-66 PMC3189133

[77] Stevens CH , Rowe D , Morel-Kopp MC , et al. (2012) Reduced T helper and B lymphocytes in Parkinson’s disease. J Neuroimmunol 252, 95–99. 22910543 10.1016/j.jneuroim.2012.07.015

[78] Li R , Tropea TF , Baratta LR , et al. (2022) Abnormal B-cell and Tfh-cell profiles in patients with Parkinson disease: a cross-sectional study. Neurol Neuroimmunol Neuroinflamm 9, e1125. 34955458 10.1212/NXI.0000000000001125 PMC8711073

[79] Orr CF , Rowe DB , Mizuno Y , et al. (2005) A possible role for humoral immunity in the pathogenesis of Parkinson’s disease. Brain 128, 2665–2674. 16219675 10.1093/brain/awh625

[80] Huang Y , Liu Z , Wang XQ , et al. (2014) A dysfunction of CD4+ T lymphocytes in peripheral immune system of Parkinson’s disease model mice. Zhongguo Ying Yong Sheng Li Xue Za Zhi 30, 567–576. 26016368

[81] Chi Y , Fan Y , He L , et al. (2011) Novel role of aquaporin-4 in CD4+ CD25+ T regulatory cell development and severity of Parkinson’s disease. Aging Cell 10, 368–382. 21255222 10.1111/j.1474-9726.2011.00677.x

[82] Chung ES , Kim H , Lee G , et al. (2012) Neuro-protective effects of bee venom by suppression of neuroinflammatory responses in a mouse model of Parkinson’s disease: role of regulatory T cells. Brain Behav Immun 26, 1322–1330. 22974722 10.1016/j.bbi.2012.08.013

[83] Ellis GI , Zhi L , Akundi R , et al. (2013) Mitochondrial and cytosolic roles of PINK1 shape induced regulatory T-cell development and function. Eur J Immunol 43, 3355–3360. 24037540 10.1002/eji.201343571 PMC4539263

[84] Duffy SS , Keating BA , Perera CJ , et al. (2018) The role of regulatory T cells in nervous system pathologies. J Neurosci Res 96, 951–968. 28488363 10.1002/jnr.24073

[85] Chen Y , Qi B , Xu W , et al. (2015) Clinical correlation of peripheral CD4+-cell sub-sets, their imbalance and Parkinson’s disease. Mol Med Rep 12, 6105–6111. 10.3892/mmr.2015.4136 26239429

[86] Saunders JA , Estes KA , Kosloski LM , et al. (2012) CD4+ regulatory and effector/memory T cell subsets profile motor dysfunction in Parkinson’s disease. J Neuroimmune Pharmacol 7, 927–938. 23054369 10.1007/s11481-012-9402-z PMC3515774

[87] Lim JJ , Reginald K , Say YH , et al. (2023) A dietary pattern for high estimated total fat amount is associated with enhanced allergy sensitization and atopic diseases among Singapore/Malaysia young Chinese adults. Int Arch Allergy Immunol 184, 975–984. 37393903 10.1159/000530948

[88] Lim JJ , Lim SW , Reginald K , et al. (2024) Association of frequent intake of trans fatty acids and saturated fatty acids in diets with increased susceptibility of atopic dermatitis exacerbation in young Chinese adults: a cross-sectional study in Singapore/Malaysia. Skin Health Dis 4, e330. 39104637 10.1002/ski2.330 PMC11297457

[89] Hantikainen E , Roos E , Bellocco R , et al. (2022) Dietary fat intake and risk of Parkinson disease: results from the Swedish National March Cohort. Eur J Epidemiol 37, 603–613. 35416636 10.1007/s10654-022-00863-8 PMC9288363

[90] Shokri-Mashhadi N , Ghiasvand R , Feizi A , et al. (2023) Association between major dietary patterns and Parkinson’s disease risk: a case–control study. Neurol Sci 45, 2003–2010. 37993683 10.1007/s10072-023-07204-x

[91] Jayaram S & Krishnamurthy PT (2021) Role of microgliosis, oxidative stress and associated neuroinflammation in the pathogenesis of Parkinson’s disease: the therapeutic role of Nrf2 activators. Neurochem Int 145, 105014. 33689805 10.1016/j.neuint.2021.105014

[92] Hauser DN & Hastings TG (2013) Mitochondrial dysfunction and oxidative stress in Parkinson’s disease and monogenic parkinsonism. Neurobiol Dis 51, 35–42. 23064436 10.1016/j.nbd.2012.10.011 PMC3565564

[93] Zimmermann M & Brockmann K (2022) Blood and cerebrospinal fluid biomarkers of inflammation in Parkinson’s disease. J Parkinsons Dis 12, S183–s200. 35661021 10.3233/JPD-223277 PMC9535573

[94] Li M , Xu T , Zheng W , et al. (2021) Triacylglycerols compositions, soluble and bound phenolics of red sorghums, and their radical scavenging and anti-inflammatory activities. Food Chem 340, 128123. 33010645 10.1016/j.foodchem.2020.128123

[95] Du YX , Chen SN , Zhu HL , et al. (2020) Consumption of interesterified medium- and long-chain triacylglycerols improves lipid metabolism and reduces inflammation in high-fat diet-induced obese rats. J Agric Food Chem 68, 8255–8262. 32643946 10.1021/acs.jafc.0c03103

[96] Sánchez Campos S , Rodríguez Diez G , Oresti GM , et al. (2015) Dopaminergic neurons respond to iron-induced oxidative stress by modulating lipid acylation and deacylation cycles. PLoS One 10, e0130726. 26076361 10.1371/journal.pone.0130726 PMC4468124

[97] Li H , Zeng F , Huang C , et al. (2024) The potential role of glucose metabolism, lipid metabolism, and amino acid metabolism in the treatment of Parkinson’s disease. CNS Neurosci Ther 30, e14411. 37577934 10.1111/cns.14411 PMC10848100

[98] Yang D , Wang X , Zhang L , et al. (2022) Lipid metabolism and storage in neuroglia: role in brain development and neurodegenerative diseases. Cell Biosci 12, 106. 35831869 10.1186/s13578-022-00828-0 PMC9277953

[99] Fang F , Zhan Y , Hammar N , et al. (2019) Lipids, apolipoproteins, and the risk of Parkinson disease. Circ Res 125, 643–652. 31382822 10.1161/CIRCRESAHA.119.314929

[100] Gómez-Soler M , Cordobilla B , Morató X , et al. (2018) Triglyceride form of docosahexaenoic acid mediates neuroprotection in experimental Parkinsonism. Front Neurosci 12, 604. 30233293 10.3389/fnins.2018.00604 PMC6127646

[101] Xie Y , Li J , Kang R , et al. (2020) Interplay between lipid metabolism and autophagy. Front Cell Dev Biol 8, 431. 32582708 10.3389/fcell.2020.00431 PMC7283384

[102] Zhang S , Peng X , Yang S , et al. (2022) The regulation, function, and role of lipophagy, a form of selective autophagy, in metabolic disorders. Cell Death Dis 13, 132. 35136038 10.1038/s41419-022-04593-3 PMC8825858

[103] Cole NB , Murphy DD , Grider T , et al. (2002) Lipid droplet binding and oligomerization properties of the Parkinson’s disease protein α-synuclein. J Biol Chem 277, 6344–6352. 11744721 10.1074/jbc.M108414200

[104] Baekelandt V , Lobbestael E , Xicoy H , et al. (2020) Editorial: the role of lipids in the pathogenesis of Parkinson’s disease. Front Neurosci 14, 250. 32265647 10.3389/fnins.2020.00250 PMC7105851

[105] Adom MA , Hahn WN , McCaffery TD , et al. (2024) Reducing the lipase LIPE in mutant *α*-synuclein mice improves Parkinson-like deficits and reveals sex differences in fatty acid metabolism. Neurobiol Dis 199, 106593. 38971480 10.1016/j.nbd.2024.106593 PMC11577057

[106] Tansey MG , Wallings RL , Houser MC , et al. (2022) Inflammation and immune dysfunction in Parkinson disease. Nat Rev Immunol (Internet) 22, 1–17. 35246670 10.1038/s41577-022-00684-6 PMC8895080

