# Supplementary material for: The protective effect of higher serum TAG (51:4) levels against Parkinson’s disease
Source: Br J Nutr. 2024 Nov 11;133(6):806–16. doi: 10.1017/S0007114524002137 (PMC12169956; doi:10.1017/S0007114524002137)
Supplement: Jing et al. supplementary material [file S0007114524002137sup001.zip › Supplementary Tables/Table.docx]

**Table I. The results of pleiotropy for MR analysis**

| **Exposure** | **Outcome** | **Egger_intercept** | **se** | **p-value** |
| --- | --- | --- | --- | --- |
| serum TGA levels | PD | -0.017 | 0.025 | 0.512 |

**Note：**

IVW, inverse-variance weighted.

PD, Parkinson’s disease.

**Table II. The results of heterogeneity for MR analysis**

| **Outcome** | **Exposure** | **Method** | **Q** | **Q-df** | **p-value** |
| --- | --- | --- | --- | --- | --- |
| PD | serum TAG levels | MR_Egger | 25.78 | 17 | 0.0786 |
| PD | serum TAG levels | IVW | 26.46 | 18 | 0.0898 |

**Note：**

IVW, inverse-variance weighted.

PD, Parkinson’s disease.

**Table III. The results of pleiotropy for MR analysis**

| **Outcome** | **Exposure** | **Egger_intercept** | **se** | **p-value** |
| --- | --- | --- | --- | --- |
| IgD- CD38- B cell %lymphocyte | serum TAG levels | -0.008 | 0.017 | 0.647 |
| Switched memory B cell %lymphocyte | serum TAG levels | -0.007 | 0.016 | 0.658 |
| IgD- CD38- B cell %B cell | serum TAG levels | -0.002 | 0.016 | 0.881 |
| IgD- CD38- B cell Absolute Count | serum TAG levels | 0.007 | 0.0187 | 0.719 |
| CD86+ plasmacytoid Dendritic Cell %Dendritic Cell | serum TAG levels | 0.024 | 0.015 | 0.139 |
| HLA DR++ monocyte %leukocyte | serum TAG levels | -0.013 | 0.015 | 0.404 |
| Resting CD4 regulatory T cell Absolute Count | serum TAG levels | 0.027 | 0.018 | 0.089 |
| Resting CD4 regulatory T cell %CD4+ T cell | serum TAG levels | 0.029 | 0.017 | 0.119 |
| CD45RA- CD4+ T cell %CD4+ T cell | serum TAG levels | -0.019 | 0.0146 | 0.213 |
| Naive CD4+ T cell Absolute Count | serum TAG levels | 0.015 | 0.015 | 0.326 |
| Naive CD4+ T cell %CD4+ T cell | serum TAG levels | 0.013 | 0.015 | 0.405 |
| Effector Memory CD4+ T cell %CD4+ T cell | serum TAG levels | -0.013 | 0.015 | 0.389 |
| Naive CD8+ T cell %CD8+ T cell | serum TAG levels | 0.011 | 0.012 | 0.351 |
| Naive CD8+ T cell %T cell | serum TAG levels | 0.008 | 0.013 | 0.549 |
| CD45RA+ CD8+ T cell %CD8+ T cell | serum TAG levels | 0.009 | 0.013 | 0.476 |
| Central Memory CD4-CD8- T cell Absolute Count | serum TAG levels | 0.011 | 0.013 | 0.414 |
| Central Memory CD4-CD8- T cell %CD4-CD8- T cell | serum TAG levels | 0.016 | 0.014 | 0.269 |
| Central Memory CD4-CD8- T cell %T cell | serum TAG levels | 0.010 | 0.014 | 0.453 |
| Naive CD4-CD8- T cell %CD4-CD8- T cell | serum TAG levels | 0.019 | 0.014 | 0.194 |
| Naive CD4-CD8- T cell %T cell | serum TAG levels | 0.019 | 0.015 | 0.198 |
| Naive CD4+ T cell %T cell | serum TAG levels | 0.012 | 0.015 | 0.423 |
| CD3- lymphocyte %lymphocyte | serum TAG levels | -0.055 | 0.038 | 0.187 |
| HLA DR+ Natural Killer Absolute Count | serum TAG levels | -0.027 | 0.016 | 0.099 |
| CD28- CD25++ CD8+ T cell %T cell | serum TAG levels | 0.049 | 0.049 | 0.369 |
| CD28- CD25++ CD8+ T cell %CD8+ T cell | serum TAG levels | -0.012 | 0.016 | 0.463 |
| CD28- CD25++ CD8+ T cell Absolute Count | serum TAG levels | -0.004 | 0.016 | 0.806 |
| CD28+ CD45RA+ CD8+ T cell %T cell | serum TAG levels | 0.007 | 0.012 | 0.562 |
| CD28+ CD45RA+ CD8+ T cell %CD8+ T cell | serum TAG levels | 0.009 | 0.012 | 0.439 |
| CD20 on IgD+ CD38- unswitched memory B cell | serum TAG levels | -0.038 | 0.021 | 0.087 |
| CD3 on CD28+ CD4-CD8- T cell | serum TAG levels | 0.006 | 0.017 | 0.705 |
| HVEM on Terminally Differentiated CD8+ T cell | serum TAG levels | 0.008 | 0.025 | 0.739 |
| CD16-CD56 on HLA DR+ Natural Killer | serum TAG levels | 0.014 | 0.017 | 0.409 |
| CD28 on secreting CD4 regulatory T cell | serum TAG levels | -0.002 | 0.017 | 0.902 |
| CD28 on CD39+ secreting CD4 regulatory T cell | serum TAG levels | -0.001 | 0.017 | 0.985 |
| CD28 on activated & secreting CD4 regulatory T cell | serum TAG levels | -0.002 | 0.017 | 0.893 |
| CD28 on CD28+ CD4+ T cell | serum TAG levels | -0.018 | 0.016 | 0.284 |
| CD28 on CD4+ T cell | serum TAG levels | -0.014 | 0.016 | 0.414 |
| CD28 on CD39+ resting CD4 regulatory T cell | serum TAG levels | 0.005 | 0.016 | 0.754 |
| CD45 on granulocyte | serum TAG levels | 0.012 | 0.018 | 0.518 |
| CD127 on CD28+ CD4+ T cell | serum TAG levels | 0.004 | 0.018 | 0.829 |
| FSC-A on myeloid Dendritic Cell | serum TAG levels | 0.016 | 0.018 | 0.350 |
| FSC-A on HLA DR+ Natural Killer | serum TAG levels | -0.025 | 0.017 | 0.152 |
| FSC-A on HLA DR+ CD8+ T cell | serum TAG levels | -0.004 | 0.016 | 0.815 |
| CD45 on CD33- HLA DR+ | serum TAG levels | -0.014 | 0.023 | 0.554 |
| SSC-A on HLA DR+ Natural Killer | serum TAG levels | -0.015 | 0.016 | 0.385 |
| HLA DR on HLA DR+ T cell | serum TAG levels | 0.011 | 0.016 | 0.503 |
| HLA DR on HLA DR+ CD4+ T cell | serum TAG levels | 0.012 | 0.017 | 0.478 |
| HLA DR on HLA DR+ Natural Killer | serum TAG levels | -0.017 | 0.017 | 0.299 |

**Table IV. The results of heterogeneity for MR analysis**

| **Outcome** | **Exposure** | **Method** | **Q** | **Q-df** | **p-value** |
| --- | --- | --- | --- | --- | --- |
| IgD- CD38- B cell %lymphocyte | serum TAG levels | MR_Egger | 21.46 | 17 | 0.206 |
|  | serum TAG levels | IVW | 21.74 | 18 | 0.244 |
| Switched memory B cell %lymphocyte | serum TAG levels | MR_Egger | 19.42 | 17 | 0.305 |
|  | serum TAG levels | IVW | 19.66 | 18 | 0.352 |
| IgD- CD38- B cell %B cell | serum TAG levels | MR_Egger | 17.95 | 17 | 0.392 |
|  | serum TAG levels | IVW | 17.97 | 18 | 0.458 |
| IgD- CD38- B cell Absolute Count | serum TAG levels | MR_Egger | 23.87 | 17 | 0.123 |
|  | serum TAG levels | IVW | 24.06 | 18 | 0.153 |
| CD86+ plasmacytoid Dendritic Cell %Dendritic Cell | serum TAG levels | MR_Egger | 11.62 | 17 | 0.823 |
|  | serum TAG levels | IVW | 14.02 | 18 | 0.728 |
| HLA DR++ monocyte %leukocyte | serum TAG levels | MR_Egger | 12.67 | 17 | 0.758 |
|  | serum TAG levels | IVW | 13.40 | 18 | 0.767 |
| Resting CD4 regulatory T cell Absolute Count | serum TAG levels | MR_Egger | 18.33 | 17 | 0.368 |
|  | serum TAG levels | IVW | 21.82 | 18 | 0.240 |
| Resting CD4 regulatory T cell %CD4+ T cell | serum TAG levels | MR_Egger | 25.14 | 17 | 0.092 |
|  | serum TAG levels | IVW | 29.12 | 18 | 0.057 |
| CD45RA- CD4+ T cell %CD4+ T cell | serum TAG levels | MR_Egger | 17.34 | 17 | 0.431 |
|  | serum TAG levels | IVW | 19.054 | 18 | 0.388 |
| Naive CD4+ T cell Absolute Count | serum TAG levels | MR_Egger | 17.90 | 17 | 0.394 |
|  | serum TAG levels | MR_Egger | 18.98 | 18 | 0.392 |
| Naive CD4+ T cell %CD4+ T cell | serum TAG levels | IVW | 20.00 | 17 | 0.274 |
|  | serum TAG levels | MR_Egger | 20.86 | 18 | 0.286 |
| Effector Memory CD4+ T cell %CD4+ T cell | serum TAG levels | IVW | 18.08 | 17 | 0.383 |
|  | serum TAG levels | MR_Egger | 18.92 | 18 | 0.397 |
| Naive CD8+ T cell %CD8+ T cell | serum TAG levels | MR_Egger | 17.73 | 17 | 0.405 |
|  | serum TAG levels | IVW | 18.69 | 18 | 0.411 |
| Naive CD8+ T cell %T cell | serum TAG levels | MR_Egger | 25.15 | 17 | 0.091 |
|  | serum TAG levels | IVW | 25.71 | 18 | 0.106 |
| CD45RA+ CD8+ T cell %CD8+ T cell | serum TAG levels | MR_Egger | 14.21 | 17 | 0.651 |
|  | serum TAG levels | IVW | 14.74 | 18 | 0.679 |
| Central Memory CD4-CD8- T cell Absolute Count | serum TAG levels | MR_Egger | 17.08 | 17 | 0.448 |
|  | serum TAG levels | IVW | 17.79 | 18 | 0.469 |
| Central Memory CD4-CD8- T cell %CD4-CD8- T cell | serum TAG levels | MR_Egger | 15.62 | 17 | 0.550 |
|  | serum TAG levels | IVW | 16.93 | 18 | 0.527 |
| Central Memory CD4-CD8- T cell %T cell | serum TAG levels | MR_Egger | 17.78 | 17 | 0.402 |
|  | serum TAG levels | IVW | 18.40 | 18 | 0.429 |
| Naive CD4-CD8- T cell %CD4-CD8- T cell | serum TAG levels | MR_Egger | 18.30 | 17 | 0.369 |
|  | serum TAG levels | MR_Egger | 20.28 | 18 | 0.317 |
| Naive CD4-CD8- T cell %T cell | serum TAG levels | IVW | 22.46 | 17 | 0.167 |
|  | serum TAG levels | MR_Egger | 24.84 | 18 | 0.129 |
| Naive CD4+ T cell %T cell | serum TAG levels | IVW | 16.29 | 17 | 0.503 |
|  | serum TAG levels | MR_Egger | 16.96 | 18 | 0.525 |
| CD3- lymphocyte %lymphocyte | serum TAG levels | IVW | 9.14 | 8 | 0.330 |
|  | serum TAG levels | MR_Egger | 11.53 | 9 | 0.241 |
| HLA DR+ Natural Killer Absolute Count | serum TAG levels | IVW | 13.42 | 17 | 0.707 |
|  | serum TAG levels | MR_Egger | 16.46 | 18 | 0.560 |
| CD28- CD25++ CD8+ T cell %T cell | serum TAG levels | MR_Egger | 4.59 | 5 | 0.467 |
|  | serum TAG levels | IVW | 5.56 | 6 | 0.473 |
| CD28- CD25++ CD8+ T cell %CD8+ T cell | serum TAG levels | MR_Egger | 20.74 | 17 | 0.238 |
|  | serum TAG levels | IVW | 21.43 | 18 | 0.258 |
| CD28- CD25++ CD8+ T cell Absolute Count | serum TAG levels | MR_Egger | 21.28 | 17 | 0.213 |
|  | serum TAG levels | IVW | 21.36 | 18 | 0.261 |
| CD28+ CD45RA+ CD8+ T cell %T cell | serum TAG levels | MR_Egger | 20.82 | 17 | 0.234 |
|  | serum TAG levels | IVW | 21.25 | 18 | 0.267 |
| CD28+ CD45RA+ CD8+ T cell %CD8+ T cell | serum TAG levels | MR_Egger | 15.25 | 17 | 0.578 |
|  | serum TAG levels | IVW | 15.87 | 18 | 0.601 |
| CD20 on IgD+ CD38- unswitched memory B cell | serum TAG levels | MR_Egger | 6.01 | 17 | 0.993 |
|  | serum TAG levels | IVW | 9.31 | 18 | 0.952 |
| CD3 on CD28+ CD4-CD8- T cell | serum TAG levels | MR_Egger | 13.36 | 17 | 0.712 |
|  | serum TAG levels | IVW | 13.50 | 18 | 0.761 |
| HVEM on Terminally Differentiated CD8+ T cell | serum TAG levels | MR_Egger | 17.52 | 17 | 0.420 |
|  | serum TAG levels | MR_Egger | 17.63 | 18 | 0.480 |
| CD16-CD56 on HLA DR+ Natural Killer | serum TAG levels | IVW | 18.20 | 17 | 0.376 |
|  | serum TAG levels | MR_Egger | 18.97 | 18 | 0.394 |
| CD28 on secreting CD4 regulatory T cell | serum TAG levels | IVW | 14.09 | 17 | 0.660 |
|  | serum TAG levels | MR_Egger | 14.11 | 18 | 0.723 |
| CD28 on CD39+ secreting CD4 regulatory T cell | serum TAG levels | IVW | 18.90 | 17 | 0.334 |
|  | serum TAG levels | MR_Egger | 18.90 | 18 | 0.398 |
| CD28 on activated & secreting CD4 regulatory T cell | serum TAG levels | IVW | 18.00 | 17 | 0.389 |
|  | serum TAG levels | MR_Egger | 18.02 | 18 | 0.454 |
| CD28 on CD28+ CD4+ T cell | serum TAG levels | MR_Egger | 15.83 | 17 | 0.536 |
|  | serum TAG levels | IVW | 17.05 | 18 | 0.519 |
| CD28 on CD4+ T cell | serum TAG levels | MR_Egger | 12.77 | 17 | 0.751 |
|  | serum TAG levels | IVW | 13.47 | 18 | 0.763 |
| CD28 on CD39+ resting CD4 regulatory T cell | serum TAG levels | MR_Egger | 13.98 | 17 | 0.668 |
|  | serum TAG levels | IVW | 14.08 | 18 | 0.724 |
| CD45 on granulocyte | serum TAG levels | MR_Egger | 22.14 | 17 | 0.179 |
|  | serum TAG levels | IVW | 22.71 | 18 | 0.202 |
| CD127 on CD28+ CD4+ T cell | serum TAG levels | MR_Egger | 19.47 | 17 | 0.302 |
|  | serum TAG levels | IVW | 19.52 | 18 | 0.360 |
| FSC-A on myeloid Dendritic Cell | serum TAG levels | MR_Egger | 12.29 | 17 | 0.782 |
|  | serum TAG levels | MR_Egger | 13.22 | 18 | 0.778 |
| FSC-A on HLA DR+ Natural Killer | serum TAG levels | IVW | 14.52 | 17 | 0.630 |
|  | serum TAG levels | MR_Egger | 16.76 | 18 | 0.539 |
| FSC-A on HLA DR+ CD8+ T cell | serum TAG levels | IVW | 11.33 | 17 | 0.839 |
|  | serum TAG levels | MR_Egger | 11.39 | 18 | 0.877 |
| CD45 on CD33- HLA DR+ | serum TAG levels | IVW | 17.16 | 16 | 0.375 |
|  | serum TAG levels | MR_Egger | 17.55 | 17 | 0.417 |
| SSC-A on HLA DR+ Natural Killer | serum TAG levels | MR_Egger | 12.25 | 17 | 0.784 |
|  | serum TAG levels | IVW | 13.05 | 18 | 0.789 |
| HLA DR on HLA DR+ T cell | serum TAG levels | MR_Egger | 15.48 | 17 | 0.561 |
|  | serum TAG levels | IVW | 15.95 | 18 | 0.596 |
| HLA DR on HLA DR+ CD4+ T cell | serum TAG levels | MR_Egger | 19.53 | 17 | 0.299 |
|  | serum TAG levels | IVW | 20.14 | 18 | 0.325 |
| HLA DR on HLA DR+ Natural Killer | serum TAG levels | MR_Egger | 11.93 | 17 | 0.805 |
|  | serum TAG levels | IVW | 13.07 | 18 | 0.787 |

**Table V. The results of pleiotropy for MR analysis**

| **Outcome** | **Exposure** | **Egger_intercept** | **se** | **p-value** |
| --- | --- | --- | --- | --- |
| Caspase 8 levels | serum TAG levels | 0.008 | 0.008 | 0.302 |
| C-C motif chemokine 20 levels | serum TAG levels | 0.001 | 0.008 | 0.860 |
| C-C motif chemokine 23 levels | serum TAG levels | 0.008 | 0.011 | 0.464 |
| Fibroblast growth factor 19 levels | serum TAG levels | -0.004 | 0.009 | 0.704 |
| Monocyte chemoattractant protein-4 levels | serum TAG levels | 0.012 | 0.008 | 0.158 |
| Macrophage inflammatory protein 1a levels | serum TAG levels | 0.009 | 0.008 | 0.249 |
| Sulfotransferase 1A1 levels | serum TAG levels | 0.003 | 0.009 | 0.729 |
| tumor necrosis factor ligand superfamily member 12 | serum TAG levels | 0.011 | 0.008 | 0.170 |

**Table VI. The results of heterogeneity for MR analysis**

| Outcome | Exposure | Method | Q | Q-df | p-value |
| --- | --- | --- | --- | --- | --- |
| Caspase 8 levels | serum TAG levels | MR_Egger | 14.02 | 18 | 0.728 |
|  | serum TAG levels | IVW | 15.15 | 19 | 0.713 |
| C-C motif chemokine 20 levels | serum TAG levels | MR_Egger | 18.07 | 18 | 0.451 |
|  | serum TAG levels | IVW | 18.11 | 19 | 0.515 |
| C-C motif chemokine 23 levels | serum TAG levels | MR_Egger | 35.78 | 18 | 0.117 |
|  | serum TAG levels | IVW | 36.89 | 19 | 0.108 |
| Fibroblast growth factor 19 levels | serum TAG levels | MR_Egger | 26.72 | 18 | 0.084 |
|  | serum TAG levels | IVW | 26.94 | 19 | 0.106 |
| Monocyte chemoattractant protein-4 levels | serum TAG levels | MR_Egger | 18.38 | 18 | 0.431 |
|  | serum TAG levels | IVW | 20.59 | 19 | 0.359 |
| Macrophage inflammatory protein 1a levels | serum TAG levels | MR_Egger | 13.59 | 18 | 0.755 |
|  | serum TAG levels | IVW | 15.02 | 19 | 0.722 |
| Sulfotransferase 1A1 levels | serum TAG levels | MR_Egger | 15.78 | 18 | 0.608 |
|  | serum TAG levels | IVW | 15.90 | 19 | 0.664 |
| tumor necrosis factor ligand superfamily member 12 | serum TAG levels | MR_Egger | 15.45 | 18 | 0.631 |
|  | serum TAG levels | IVW | 17.49 | 19 | 0.557 |

**Table VII. The results of pleiotropy for MR analysis**

| **Outcome** | **Exposure** | **Egger_intercept** | **se** | **p-value** |
| --- | --- | --- | --- | --- |
| PD | IgD- CD38- B cell %lymphocyte | 0.007 | 0.013 | 0.148 |
| PD | Resting CD4 regulatory T cell %CD4+ T cell | -0.009 | 0.013 | 0.467 |

**Table VIII. The results of heterogeneity for MR analysis**

| **Outcome** | **Exposure** | **Method** | **Q** | **Q-df** | **p-value** |
| --- | --- | --- | --- | --- | --- |
| PD | IgD- CD38- B cell %lymphocyte | MR_Egger | 21.05 | 26 | 0.739 |
| PD |  | IVW | 25.36 | 27 | 0.554 |
| PD | Resting CD4 regulatory T cell %CD4+ T cell | MR_Egger | 23.99 | 30 | 0.772 |
| PD |  | IVW | 24.53 | 31 | 0.788 |
